# The Roles of Macrophage Lineage Cells (MLCs) in Brain Aging

**DOI:** 10.1002/cns.70873

**Published:** 2026-04-12

**Authors:** Qin Qin, Liubin Zhang, Manning Guo, Danli Lu, Yuxin Liu, Zihong Wang, Mengyan Hu, Shisi Wang, Xinmei Kang, Haotong Yi, Wei Qiu, Zhengqi Lu, Wei Cai

**Affiliations:** ^1^ Department of Neurology, Mental and Neurological Disease Research Center The Third Affiliated Hospital of Sun Yat‐Sen University Guangzhou China; ^2^ Guangdong Provincial Key Laboratory of Brain Function and Disease Guangzhou China

**Keywords:** border‐associated macrophage, brain aging, microglia, monocyte‐derived macrophage

## Abstract

**Background:**

Brain aging poses a major public health challenge and is the primary risk factor for neurodegenerative diseases. Macrophage lineage cells (MLCs) have emerged as pivotal mediators of brain aging. While fundamental to central nervous system (CNS) homeostasis through their scavenging, detoxification, and neurotrophic functions, their transition to a senescent state is a primary driver of pathology. This shift is marked by a loss of clearance capacity and the adoption of a pro‐inflammatory senescence‐associated secretory phenotype (SASP).

**Objectives:**

Here, we summarize the distinct and cooperative roles of MLC subsets in brain aging. We examine the key molecular drivers of MLCs senescence and detail how subset‐specific dysfunction contributes to the propagation of cellular aging and related neuropathology. Finally, we evaluate current and emerging therapeutic strategies that target MLCs senescence.

**Conclusion:**

We conclude by proposing a multidimensional management framework for brain aging. This framework positions MLCs as a central therapeutic hub, integrating advanced diagnostics and stratified interventions to preserve brain health and mitigate neurodegenerative pathology.

## Graphical abstract

This review highlights the critical role of macrophage lineage cells (MLCs), including microglia, border‐associated macrophages (BAMs), and CNS‐infiltrating monocyte‐derived macrophages (MDMs), in brain aging. It explores the drivers and propagation of MLCs senescence and discusses potential therapeutic strategies targeting MLCs senescence.

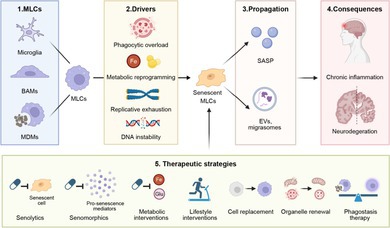

## 1 Introduction

Brain aging, characterized by a progressive decline in cognitive and memory functions, represents one of the 21st century's most formidable public health challenges. It diminishes the quality of life for the aged population and stands as the principal risk factor for neurodegenerative disorders, including Alzheimer's disease (AD) and Parkinson's disease (PD). With global dementia cases projected to exceed 150 million by 2050 [1], elucidating the mechanisms that drive this decline is a global priority. Neuroimmunological studies have dismantled the long‐held paradigm of central nervous system (CNS) “immune privilege”. This shift has solidified the concept of “inflammaging”, a chronic, low‐grade inflammatory state, as a fundamental driver of brain aging, positioning CNS‐resident immune cells as its central regulators [2, 3].

**FIGURE 1:**
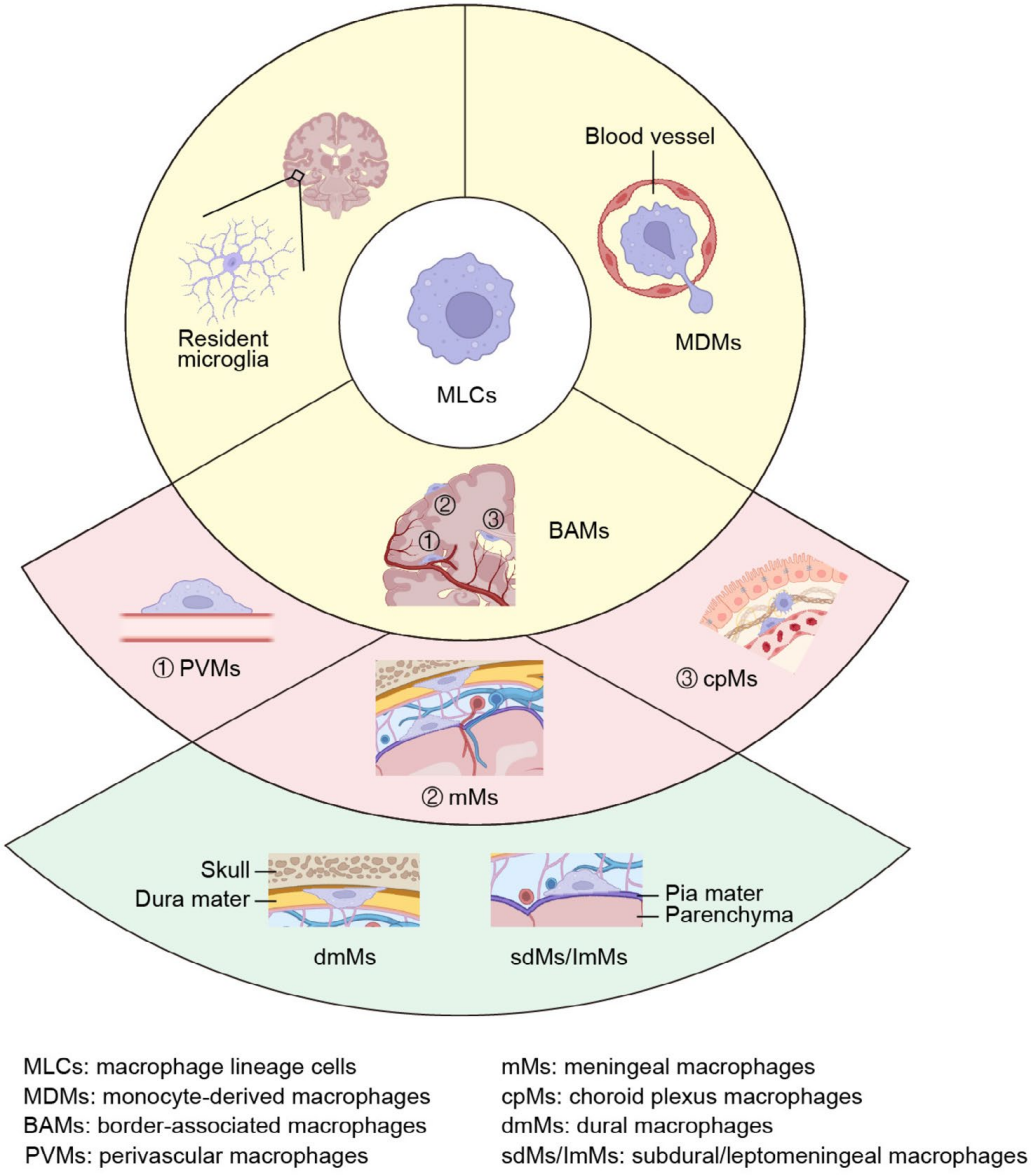
Major components of brain macrophage lineage cells (MLCs). The central nervous system (CNS) MLCs consist of parenchymal microglia, border‐associated macrophages (BAMs), and monocyte‐derived macrophages (MDMs). BAMs are strategically positioned at CNS interfaces and include perivascular macrophages (PVMs), meningeal macrophages, and choroid plexus macrophages (cpMs). The meningeal macrophage population is composed of dural macrophages (dmMs) and subdural/leptomeningeal macrophages (sdMs/lmMs). During pathological states, circulating monocytes infiltrate the CNS and give rise to MDMs.

Among the diverse CNS immune cell populations, macrophage lineage cells (MLCs), encompassing brain‐resident microglia, border‐associated macrophages (BAMs), and CNS‐infiltrating monocyte‐derived macrophages (MDMs), have emerged as a pivotal focus in aging research (Figure 1) [4]. The indispensable role of MLCs in this process is underscored by several key characteristics: (1) a scavenging function that acts as the core defense against toxic accumulation, as MLCs are among the most critical phagocytes responsible for clearing metabolic waste, apoptotic cells, and misfolded protein aggregates; (2) robust internal detoxification machinery, including the lysosomal pathway and antioxidant systems, which represent a fundamental pillar of CNS homeostasis by neutralizing the cytotoxic byproducts of metabolism and phagocytosis, meaning its failure significantly contributes to a toxic environment that accelerates brain aging; (3) a dynamic secretome that coordinates the microenvironment, which in homeostasis involves the release of neurotrophic factors, but in senescence, shifts to a pathological pro‐inflammatory profile (the senescence‐associated secretory phenotype, SASP) that actively transmits senescence signals to adjacent cells; and (4) a potent regenerative potential through self‐renewal and peripheral replenishment, which endows MLCs with high plasticity and substantial regenerative capacity, conferring a unique therapeutic advantage over post‐mitotic neurons [5, 6]. While some evidence suggests that MLC dysfunction may be reversible [7, 8], the extent to which established senescence can be reversed remains debated; nonetheless, even partial restoration of MLC function holds significant therapeutic promise.

In this review, we analyze the distinct and cooperative roles of MLC subsets in brain aging, examining their developmental origins, functional specializations, and interaction networks. We explore the key molecular drivers of MLCs senescence, from oxidative stress and mitochondrial dysfunction to epigenetic reprogramming, and detail how subset‐specific senescence contributes to synaptic loss, persistent neuroinflammation, and cognitive decline. Finally, we summarize current and emerging therapeutic strategies targeting MLCs senescence, discussing their potential to promote healthy brain aging and ameliorate the burden of neurodegenerative disease.

## 2 The Heterogeneous Family of MLCs in the CNS

MLCs comprise a highly heterogeneous compartment that plays essential roles in preserving CNS homeostasis and orchestrating brain function across the lifespan. This cellular diversity includes microglia, BAMs, and MDMs, each constituting a distinct yet interconnected component of the CNS immune milieu [9] (Table 1).

**TABLE 1:** Comparative characteristics of MLCs.

	Microglia	BAMs					MDMs
		PVMs	sdMs	dmMs	cpMs		
					cp^hi^Ms, cp^lo^Ms	cp^epi^Ms	
Localization	Brain and spinal cord parenchyma	Perivascular spaces	Subdural meninges	Dura mater	Choroid plexus		Dura mater and choroid plexus under physiological conditions; parenchymal infiltration under pathological conditions
Prenatal origin	Early EMPs in the extraembryonic yolk sac during primitive hematopoiesis	Early EMPs in the extraembryonic yolk sac during primitive hematopoiesis					HSCs emerging in the AGM and subsequently migrating to the fetal liver during definitive hematopoiesis
Postnatal maintenance	Self‐maintain through clonal proliferation	Self‐maintain and infiltration of sdMs	Self‐maintain	Differentiated from HSC‐derived monocytes supplied in the skull and vertebral bone marrow		Self‐maintain	Bone marrow‐derived HSCs
Gene markers	*P2ry12*, *Tmem119*, *Sall1, Hexb, Siglech*	*Ms4a7, Apoe, Lyz2, Mrc1, CD163, Lyve1*	*Ms4a7, Apoe, Lyz2, Lyve1, P2rx7, Egfl7*	*Ms4a7, Apoe, Lyz2, Pla2g2d, Ccl8*	*Ms4a7, Apoe, Lyz2, Lilra5, Ttr*	*Ms4a7, Apoe, Lyz2, Cst7, Gm1673, Clec7a*	*Ccl2, Ccr2, Adgre1*
Aging‐associated phenotypes	Dystrophic morphology, Impaired phagocytosis and debris clearance, Aberrant synaptic pruning, Lipofuscin accumulation and lysosomal dysfunction, Lipid droplet‐associated phenotype (LDAM), Iron dyshomeostasis and oxidative stress	Reduced MMP secretion and ECM remodeling, Impaired glymphatic‐meningeal lymphatic clearance, Declined immune‐gating function					Epigenetically primed pro‐inflammatory state, Sustained inflammatory signaling, Impaired phagocytic capacity
Key reference	[9, 10, 11, 12, 13, 14, 15, 16, 17, 18]	[9, 10, 19, 20, 21, 22, 23, 24]					[9, 25, 26, 27, 28]

Abbreviations: AGM, aorta‐gonad‐mesonephros; BAMs, border‐associated macrophages; cp^epi^Ms, epiplexus macrophages; cp^hi^Ms, MHCII‐high choroid plexus macrophages; cp^lo^Ms, MHCII‐low choroid plexus macrophages; cpMs, choroid plexus macrophages; dmMs, dural macrophages; EMPs, erythro‐myeloid progenitors; HSCs, hematopoietic stem cells; LDAM, lipid‐droplet‐accumulating microglia; MDMs, monocyte‐derived macrophages; MLCs, macrophage lineage cells; MMP, matrix metalloproteinases; PVMs, perivascular macrophages; sdMs, subdural macrophages.

### 2.1 Microglia: The Resident Macrophages of the Brain Parenchyma

**FIGURE 2:**
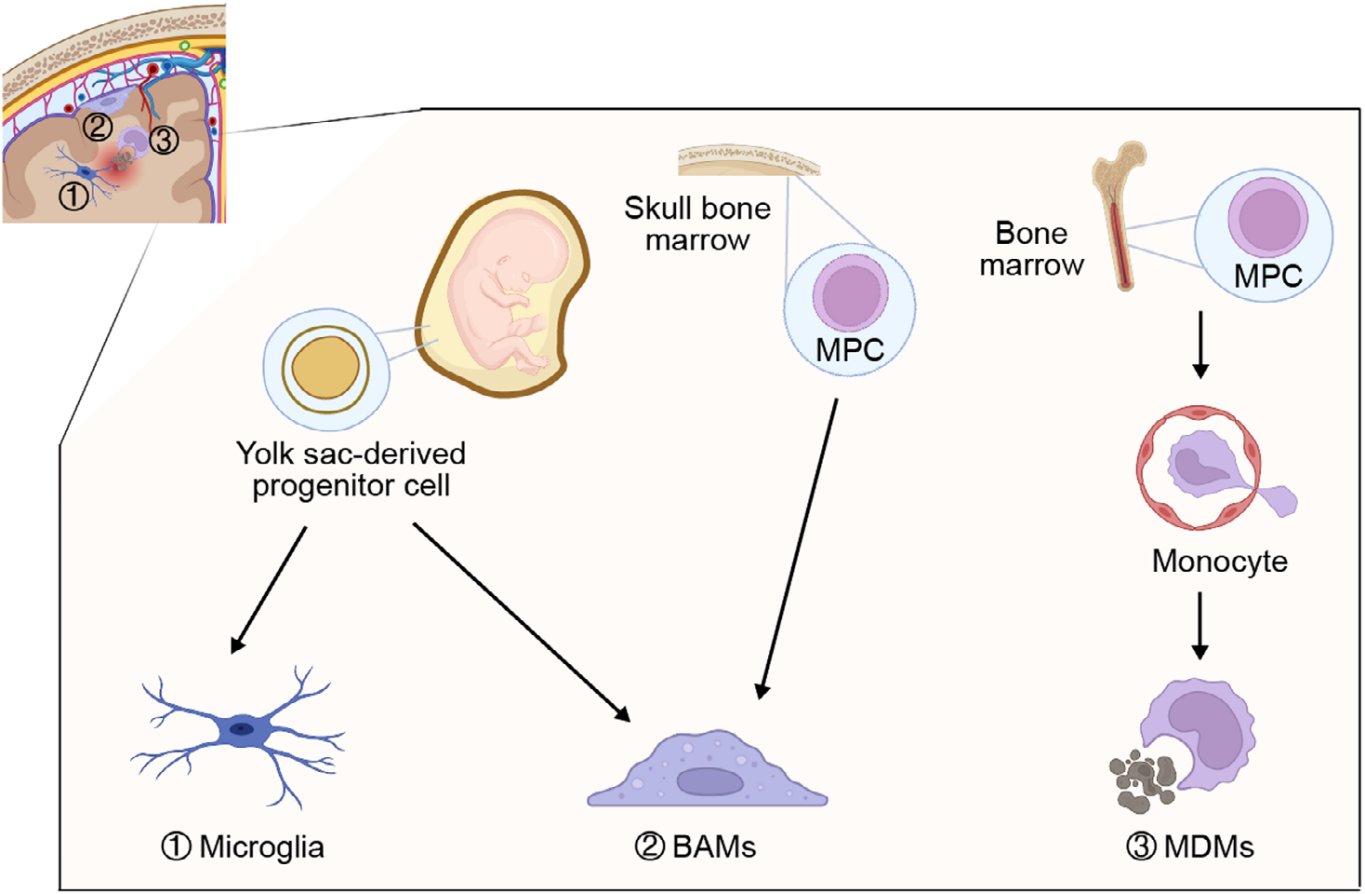
Origins and maintenance of brain MLCs. CNS MLCs have distinct developmental origins and maintenance modes. Microglia arise from yolk sac progenitors and are sustained via clonal proliferation. BAMs also originate from yolk sac progenitors and are maintained by self‐renewal, with contributions from skull bone marrow‐derived myeloid progenitors. MDMs are generated from bone marrow hematopoietic stem cells and infiltrate the CNS during disease or aging.

Microglia populate the CNS parenchyma and adopt a highly ramified morphology under physiological conditions. They originate from yolk sac‐derived erythromyeloid precursors that enter the developing brain via the nascent circulatory system at approximately 4.5 weeks of human gestation (Figure 2) [10]. During colonization, these progenitors progressively acquire a microglial identity characterized by the induction of lineage‐defining markers such as *P2ry12, Tmem119 and Sall1* [9, 11, 12]. Once established, microglia are maintained through local self‐renewal, with minimal contribution from circulating monocytes under homeostatic conditions [10, 13, 29, 30].

Microglia serve as pivotal sentinels of the brain, safeguarding homeostasis, modulating synaptic circuits and preserving functional integrity. Under physiological conditions, they continuously survey the parenchymal environment, decoding fluctuations in synaptic activity and sensing local molecular cues [31, 32]. In parallel, microglia conduct targeted phagocytosis of apoptotic cells, myelin debris and protein aggregates, thereby sustaining neural circuit stability [33]. During development, they contribute to synaptic refinement by selectively engulfing superfluous synapses in a noninflammatory manner [34]. Beyond these clearance functions, microglia also actively support neuronal health by secreting trophic factors that promote neuronal survival and synaptic integrity, an immunotrophic function that operates under both physiological and pathological conditions [6, 35, 36]. These functions are reinforced through intricate signaling networks with other brain cells, which enable microglia to modulate synaptic remodeling, coordinate glial activity and maintain an immunologically balanced CNS milieu [37, 38].

Accumulating evidence underscores the profound regional and temporal heterogeneity of microglia. Although originating from a common embryonic lineage, microglia progressively diverge to acquire region‐specific phenotypes, which are dynamically reshaped in response to pathological stimuli [14, 39, 40, 41]. This functional specialization implies that different brain regions may harbor distinct vulnerabilities to aging, largely shaped by their unique microglial profiles. Separately, the observation that certain fetal microglial subsets exhibit transcriptional signatures reminiscent of adult disease‐associated states suggests that chronic stress or aging might trigger a dysfunctional reprogramming [42, 43]. Delineating the molecular logic governing both this regional heterogeneity and the potential for pathological reprogramming is pivotal. It may reveal why specific brain regions are resilient while others are susceptible, offering new entry points for antiaging interventions.

### 2.2 BAMs: Sentinels of the CNS Interfaces

BAMs constitute pivotal immune sentinels that preserve homeostasis and orchestrate defense across CNS border interfaces. They originate from yolk sac‐derived progenitors that infiltrate the developing CNS via the nascent circulation at approximately 4.5 weeks postconception in humans, a developmental timeline that largely overlaps with the entry of microglial progenitors [9, 10]. Upon entry into CNS interface tissues, these progenitors differentiate into BAMs and progressively acquire their lineage‐specific identity through the upregulation of markers such as *Siglec1, Mrc1 and Lyve1* [44]. As development advances, BAMs diversify into anatomically distinct subsets, including meningeal macrophages (mMs), perivascular macrophages (PVMs), and choroid plexus macrophages (cpMs). Within the meninges, further compartmentalization gives rise to dural macrophages (dmMs) and subdural/leptomeningeal macrophages (sdMs/lmMs) [19, 25]. Once established, BAMs in permissive border territories, such as dmMs and cpMs, undergo gradual turnover and are progressively replenished by monocyte‐derived macrophages, which ultimately dominate these niches [20]. By contrast, BAMs residing in restricted microenvironments, including sdMs and PVMs, are largely sustained through local self‐renewal, with negligible contribution from circulating monocytes [9, 10, 21, 22].

BAMs fulfill multifaceted roles in regulating CNS immunity and interfacial tissue function. While all BAMs retain both phagocytic and antigen‐presenting capabilities, dmMs and cpMs, which exhibit an MHCII^hi^ phenotype, specialize in antigen presentation, whereas sdMs and PVMs, which adopt an MHCII^low^ phenotype, are primarily dedicated to phagocytosing metabolic waste [45, 46]. Additionally, PVMs are involved in maintaining blood–brain barrier (BBB) integrity and modulating its permeability, thereby safeguarding CNS immune privilege [47, 48]. Furthermore, through regulation of the perivascular milieu, PVMs together with sdMs facilitate cerebrospinal fluid (CSF) dynamics and glymphatic drainage, which play a critical role in metabolic waste removal and long‐term brain homeostasis [23, 49]. Also, these cells preserve neuronal function by producing trophic factors such as vascular endothelial growth factor (VEGF) and mesencephalic astrocyte‐derived neurotrophic factor (MANF) that directly support neurovascular unit (NVU) integrity [50].

Current evidence suggests that PVMs contribute to extracellular matrix (ECM) turnover by producing or activating matrix metalloproteinases (MMPs), thereby modulating arterial stiffness, a hallmark of vascular aging [19]. However, the mechanisms by which PVMs sense ECM damage or altered vascular tension, as well as how they coordinate with endothelial cells to regulate vascular dynamics, remain unresolved. These gaps raise the possibility that restoring PVMs‐mediated ECM clearance during aging could represent a viable strategy to counteract vascular decline.

### 2.3 MDMs: Peripheral Reinforcement in Health and Diseases

MDMs function as pivotal immune effectors in the CNS under pathological conditions. They arise from bone marrow hematopoietic stem cells (HSCs) (Figure 2) and differentiate into two principal monocyte subsets: classical CD14^+^ monocytes, which are capable of transmigrating into tissues, and nonclassical CD16^+^ monocytes, which patrol the vascular endothelium [26]. During CNS injury or inflammation, CD14^+^ monocytes infiltrate the parenchyma and differentiate into MDMs, acquiring the ability to secrete pro‐inflammatory mediators and thereby amplifying local immune activation [25, 27, 28, 41]. In parallel, MDMs adopt anti‐inflammatory phenotypes to restrain excessive leukocyte recruitment, promote tissue remodeling through phagocytic clearance, and support neuronal survival via paracrine release of proregenerative factors [51, 52, 53].

Recent studies indicate that, during aging, a subset of MDMs acquires a partial microglia‐like transcriptional profile, suggesting their potential for long‐term persistence within the CNS [41]. This raises the possibility that age‐related dysfunction may, in part, stem from the limited capacity of MDMs to functionally replace senescent or compromised resident macrophages. Deciphering the contribution of MDMs to the aging CNS is therefore essential for elucidating mechanisms of neuroimmune senescence. Furthermore, strategies aimed at reprogramming MDMs toward a resident macrophage‐like phenotype may offer a potential avenue for mitigating age‐associated neurodegeneration.

## 3 Molecular Hallmarks and Propagation of MLCs Senescence

The understanding of MLCs in brain aging has evolved from a model of “chronic activation” to one of functional senescence. Historically, seminal work by Streit et al. provided in vivo evidence of microglial dystrophy in the aged human brain [15], establishing a paradigm shift toward viewing these cells as functionally exhausted rather than merely overactive.

Molecularly, senescent MLCs are often characterized by stable cell‐cycle arrest mediated by p16^INK4a^/p21^WAF1^, increased SA‐β‐gal activity, and SASPs. However, these markers are not entirely specific as p16/p21 can be transiently upregulated in activated microglia without permanent cell‐cycle exit, and SASPs overlap with general pro‐inflammatory mediators [54, 55, 56]. Therefore, in this review, we operationally define MLC senescence as a state of stable functional decline accompanied by multiple concurrent hallmarks, rather than relying on any single marker. Where possible, we distinguish between replicative senescence driven by telomere attrition and stress‐induced premature senescence triggered by oxidative/phagocytic stress.

**FIGURE 3:**
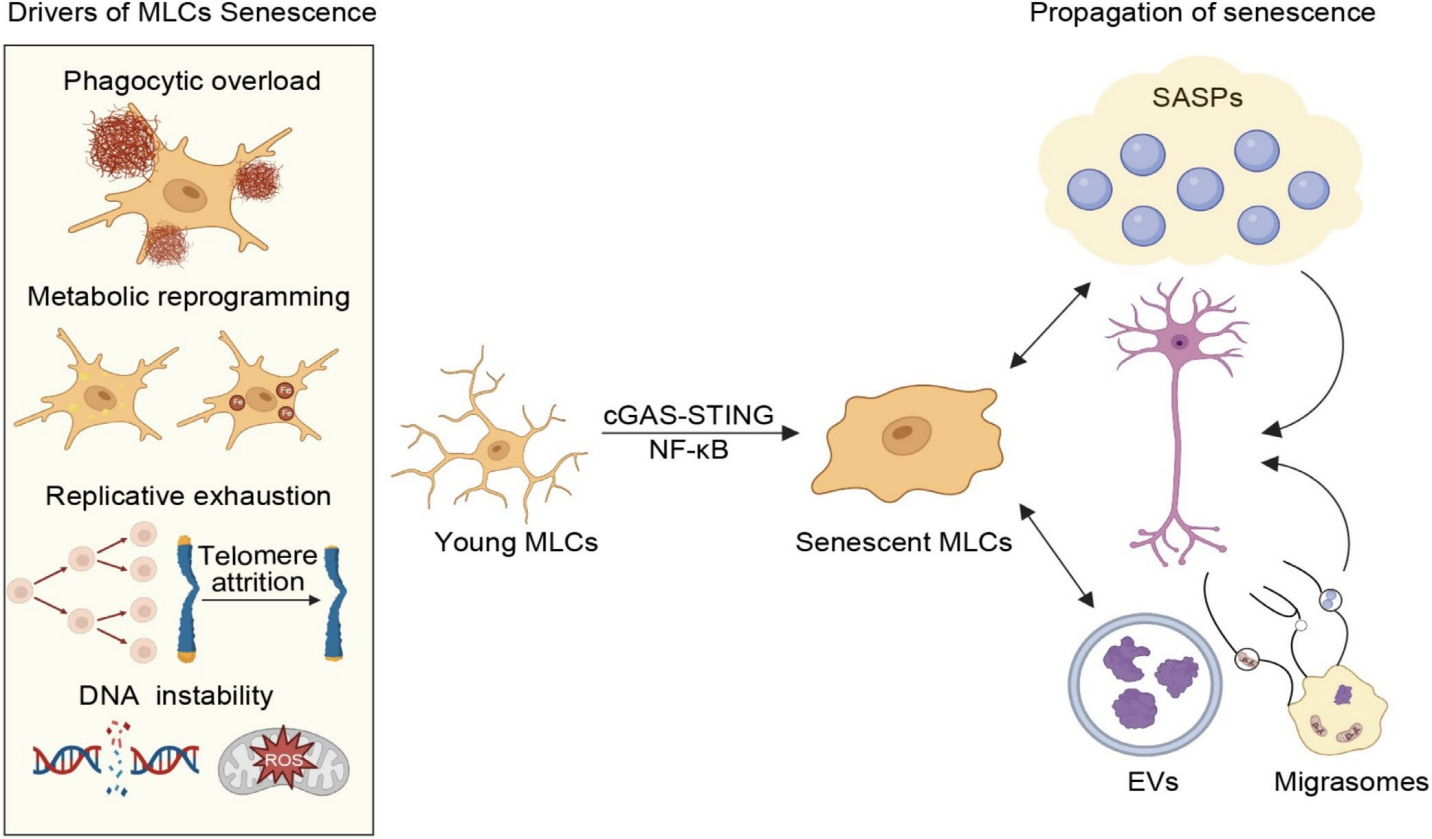
Drivers of MLCs senescence and the subsequent pro‐senescent products. Multiple drivers, including phagocytic overload, metabolic reprogramming, replicative exhaustion, and DNA instability, converge on pathways such as cGAS‐STING and NF‐κB, ultimately leading to cellular senescence in MLCs. The senescent cells then propagate damage through two primary mechanisms: the secretion of a senescence‐associated secretory phenotype (SASP) and the release of extracellular vesicles (EVs) and migrasomes.

In this section, we examine the primary drivers of these states, the mechanisms of their propagation, and their collective impact on brain aging (Figure 3).

### 3.1 Drivers of MLCs Senescence

With advancing age or under pathological conditions, MLCs may undergo cellular senescence, a state of irreversible cell cycle arrest accompanied by profound alterations in cellular functions. Cellular senescence can be triggered by a variety of intrinsic and extrinsic stressors, including excessive phagocytic burden, replicative exhaustion, and genomic instability. These factors often act in concert, reinforcing one another to accelerate the senescence process and ultimately compromising the normal function of the CNS.

#### 3.1.1 Phagocytic Overload and Lysosomal Stress

Chronic exposure to and clearance of metabolic waste leads to lysosomal dysfunction, a primary trigger of MLCs senescence [57]. The overwhelming phagocytic load exceeds the lysosome's degradation capacity, leading to the accumulation of undigested, auto‐fluorescent material (lipofuscin) within lysosomal inclusions. This buildup compromises lysosomal integrity, promotes the generation of reactive oxygen species (ROS), and provides a persistent internal stress signal that activates the cellular senescence program. The triggering receptor expressed on myeloid cells 2 (TREM2)‐apolipoprotein E (APOE) axis is central to this pathway. TREM2 is essential for sensing and initiating the clearance of lipids, toxic aggregates, and myelin fragments. While this signaling is protective in acute settings, chronic overload from persistent debris (e.g., myelin fragments) transforms this response. Instead of promoting resolution, this incessant and maladaptive TREM2 activation, coupled with the overwhelming phagocytic burden, may exhausts the cell and exacerbates the lysosomal dysfunction, potentially contributing to the transition of MLCs into a senescent state [16, 58, 59].

#### 3.1.2 Metabolic Reprogramming

Given the critical roles of phagocytosis and lysosomal function in MLCs metabolism, excessive phagocytic burden and lysosomal stress can disrupt their metabolic homeostasis, thereby promoting cellular senescence.

Senescent MLCs display distinct metabolic reprogramming, notably lipid droplet accumulation and iron dyshomeostasis, which drives a pro‐inflammatory phenotype [14, 60]. For instance, prostaglandin E2 (PGE2), elevated in aging and neurodegenerative diseases, orchestrates metabolic reprogramming in MLCs by channeling glucose into glycogen storage. This suppresses both glycolytic flux and mitochondrial respiration, creating an energy‐deficient state that fuels maladaptive inflammation [7].

This reprogramming manifests as lipid droplet‐associated microglia (LDAM), which exhibit marked phagocytic impairment and increased production of ROS and pro‐inflammatory cytokines. Such metabolic reprogramming may also compromise the energy‐demanding synthesis of trophic factors, further diminishing the neuro‐supportive capacity of MLCs [14]. Concurrently, disruption of cerebral iron homeostasis also contributes to MLCs dysfunction [61, 62]. Although microglia are central to maintaining iron balance, age‐related iron overload shifts them from a protective to a pro‐inflammatory state. This excess iron promotes microglial oxidative stress by elevating ROS, ultimately resulting in cellular senescence [17]. This iron‐induced oxidative stress is thought to amplify neuroinflammation by activating signaling cascades such as the nuclear factor κ‐light‐chain‐enhancer of activated B cells (NF‐κB) pathway, linking iron metabolism to inflammatory phenotypes and ferroptosis susceptibility.

#### 3.1.3 Sustained Proliferation and Replicative Exhaustion

In response to chronic neuroinflammatory stimuli, MLCs leverage their mitotic capacity to undergo sustained proliferation. This excessive division, however, leads to telomere attrition and replicative exhaustion. As telomeres shorten beyond a critical threshold, MLCs enter a replicative senescent state characterized by upregulation of cyclin‐dependent kinase inhibitors such as cyclin‐dependent kinase inhibitor 2A (p16^INK4a^) [63, 64]. Importantly, animal studies using genetic ablation of p16^INK4a+^ senescent cells, including microglia, have shown that their removal alleviates age‐associated neuropathology, including neuroinflammation, Aβ deposition, tau pathology, and cognitive decline, indicating that p16^INK4a+^ senescent MLCs are not merely correlates of aging but are key pathological drivers of neurodegeneration.

However, p16^INK4a^ expression alone does not define replicative senescence [56, 64]. For instance, disease‐associated microglia (DAM) upregulate p16^INK4a^ and other senescence‐associated genes, yet they may represent a transient activation state rather than true terminal senescence [40, 65]. This underscores the need for multimarker approaches when identifying senescent MLCs.

#### 3.1.4 Genomic and Mitochondrial DNA Instability

Genomic integrity is constantly challenged by diverse stressors, leading to cumulative DNA damage. Crucially, this instability applies to both the nuclear genome and, perhaps more potently, mitochondrial DNA (mtDNA), which is highly vulnerable to damage and lacks robust repair mechanisms. This damage activates chronic DNA damage responses (DDR) and leads to the accumulation of cytosolic DNA fragments, including leaked mtDNA.

These cytosolic fragments are potent danger signals recognized by the cyclic GMP‐AMP synthase (cGAS)‐stimulator of interferon genes (STING) signaling cascade. Activation of cGAS‐STING represents a major driver of the SASP and the type I interferon (IFN‐I) signaling. This IFN‐I signature is a hallmark of senescent microglia, directly linking DNA instability to chronic, noninfectious neuroinflammation. While persistent DDR also engages canonical tumor suppressor pathways (like p53/p21) to enforce cell cycle arrest, its activation of the cGAS‐STING‐IFN axis represents a distinct mechanism by which DNA instability drives the proaging inflammatory state [66].

Collectively, these universal drivers of senescence culminate in distinct phenotypic manifestations across MLC subsets, as detailed by high‐dimensional single‐cell mapping [25]. In parenchymal microglia, aging induces a transition from a homeostatic state toward a “reactive” phenotype, characterized by the upregulation of activation markers and a shift in their functional signature. In contrast, BAMs follow a unique senescent trajectory at CNS interfaces; while all BAM subsets (meningeal, perivascular, and choroid plexus) constitutively express CD206, aging specifically leads to the enrichment of a CD38^+^MHCII^+^ subset, indicating a metabolic‐inflammatory reprogramming at the brain's boundaries. Furthermore, the aged CNS environment exhibits an increased presence of MDMs with a CD45^hi^Ly6C^hi^ signature, which are more influenced by peripheral systemic milieu than intrinsic CNS cues. These subtype‐specific senescent features form a complex cellular landscape that collectively accelerates brain aging.

### 3.2 Mechanisms of MLCs Senescence in the Aging Brain

The transition of MLCs from functional competence to senescence is driven by both a conserved molecular program shared across all subsets and by unique, cell type‐specific mechanisms (Figure 3).

#### 3.2.1 Shared Molecular Mechanisms Underlying MLCs Senescence

Across MLC populations in the CNS, including resident microglia, BAMs and MDMs, a conserved molecular program drives the transition from functional competence to senescence. Rather than relying on a solitary marker, senescence in MLCs is best defined by integrated molecular and functional signatures. Key features include stable cell‐cycle arrest driven by p16^INK4a^ and p21^CIP1^ expression, alongside lysosomal lipofuscin accumulation, a hallmark of chronic metabolic stress. Concurrently, the emergence of a senescence‐associated secretory phenotype enables these cells to modulate the local microenvironment via pro‐inflammatory and proteolytic factors [63]. Although these core pathways are shared, the phenotypic manifestations and functional outputs differ across MLC subsets, dictated by their unique origins and homeostatic turnover capacities.

Key shared modules include: (1) DDR activation: Genomic instability, telomere shortening, and persistent DNA double‐strand breaks activate the DDR, leading to the upregulation of canonical cell‐cycle inhibitors such as p16^INK4a^ and resulting in stable proliferative arrest [67, 68]. (2) Organelle dysfunction: Mitochondrial impairment and lysosomal or autophagic defects cause the accumulation of ROS and cytosolic nucleic acids. These stressors activate innate immune sensors, including the cGAS‐STING pathway, thereby sustaining IFN‐I and NF‐κB signaling that drive SASP [66, 69, 70]. (3) Epigenetic and enhancer remodeling: Chronic stress and inflammatory stimuli reshape chromatin architecture, often through bromodomain‐containing protein 4 (BRD4)‐dependent super‐enhancer activation. This remodeling establishes a transcriptional program that promotes SASP expression, enhances inflammatory cytokine release, and reduces cellular homeostasis and clearance capacity [71, 72]. (4) Metabolic reprogramming: Senescent MLCs shift from oxidative phosphorylation to glycolysis and display abnormal lipid metabolism [73]. Combined with inefficient removal of damaged components, these changes lock cells into a high‐SASP, low‐clearance, and trophically impaired state that reinforces senescence propagation in a paracrine manner.

Collectively, these interconnected mechanisms convert initial insults, such as debris overload, inflammation, or metabolic stress, into a stable senescent phenotype defined by sustained secretory activity, functional decline, and resistance to reversal.

#### 3.2.2 Subtype‐Specific Mechanisms

In addition to these shared molecular pathways, microglia, BAMs, and MDMs also exhibit cell type‐specific mechanisms of senescence.

Microglial senescence is further accelerated by LDAM, which arises from myelin debris overload and dysfunction of the TREM2‐APOE lipid clearance pathway. This disturbance promotes cholesteryl ester buildup, oxidative stress, and lysosomal dysfunction [14, 74]. At the same time, persistent activation of inflammasome components such as NOD‐, LRR‐ and pyrin domain‐containing protein 3 (NLRP3) amplifies IL‐1β and IL‐18 production, reinforcing SASP signaling and maintaining chronic neuroinflammation [75].

Besides, senescence in BAMs is functionally defined by the compromise of interface gating. Their continuous high‐capacity clearance function exposes them to sustained lysosomal stress. Critically, they exhibit a specific downregulation of lymphatic‐associated markers (e.g., LYVE1) and MMPs. This molecular shift transforms BAMs from active gatekeepers into passive obstructions, thereby compromising glymphatic exchange and waste clearance at CNS borders [23, 46].

MDMs undergo senescence through the combined influence of systemic aging and local neuroinflammatory cues. MDMs represent a unique case where senescence is largely “imported” rather than locally acquired. Circulating monocytes from senescent hematopoietic stem and progenitor cells (HSPCs) carry persistent epigenetic marks of “trained immunity”, predisposing them to inflammatory and inefficient clearance states [76, 77]. Within the CNS, these cells encounter oxidative metabolites, misfolded proteins, and an inflammatory environment that activate NF‐κB/signal transducer and activator of transcription (STAT) pathways, DNA damage responses, inflammasomes, and the cGAS‐STING axis, particularly under conditions of mitophagy and lysosomal failure. Thus, their senescent phenotype is driven by systemic bioenergetic deficits, making them distinct effectors of “inflammaging” that exacerbate neuroinflammation upon infiltration.

### 3.3 Propagation of Senescence by MLCs

While the previous section detailed how MLCs become senescent, an even more severe consequence is their ability to actively propagate this state. MLCs can transmit senescent signals to neighboring cells through intercellular communication mechanisms, primarily SASP, extracellular vesicles (EVs), and migrasomes.

#### 3.3.1 The Senescence‐Associated Secretory Phenotype (SASP)

Through activation of key signaling pathways such as NF‐κB, inflammasomes, and the cGAS‐STING axis, senescent MLCs release a potent cocktail of inflammatory mediators that propagate senescence to neighboring cells, namely SASPs [78, 79].

SASP serves as the primary mechanism for paracrine signaling by senescent cells, enabling them to influence a broad range of local and systemic biological processes within their tissue microenvironment. The SASP comprises a complex mixture of pro‐inflammatory cytokines, chemokines, growth factors, and proteases that can drive or exacerbate age‐related pathologies, including both degenerative and proliferative disorders [80, 81]. For example, in the CNS, SASP factors secreted by senescent microglia directly impair the function of neighboring cells. It is reported that senescent‐like microglia accumulate in demyelinated lesions and secrete CCL11/Eotaxin‐1, which inhibits oligodendrocyte maturation and limits remyelination [82].

#### 3.3.2 EVs and Migrasomes

In addition to SASP, increased production of age‐associated EVs and migrasomes by senescent MLCs is evident. Release of bioactive cargo within EVs and migrasomes serves as a noncanonical transmitter of prosenescent signals throughout the CNS.

EVs serve as a key vector for transmitting complex prosenescent signals. By carrying specific cargos, such as miRNAs, damaged mitochondrial components, and misfolded proteins, the uptake of MLCs‐derived EVs can reprogram recipient cells toward a senescent‐like phenotype. This process impairs proteostasis, triggers mitochondrial dysfunction and oxidative stress, and activates DDR, collectively leading to the upregulation of cell‐cycle inhibitors like p16^INK4a^ and sustained growth arrest. The specific mechanisms underlying this EVs‐mediated reprogramming include: (1) miRNA transfer: EVs enriched in inflammation‐associated miRNAs (e.g., miR‐21, miR‐146a) modulate gene expression in recipient glia and neurons, causing genomic instability and reinforcing the SASP network [83, 84]. (2) Mitochondrial cargo: MLCs‐derived mitochondrial EVs (mitoEVs) transport oxidized proteins and mtDNA, which act as damage‐associated molecular patterns (DAMPs). This cargo induces ROS accumulation, activates DDR, and triggers cGAS‐STING‐NF‐κB signaling in recipient cells, further amplifying senescence [85]. (3) Proteopathic stress: Microglia‐derived EVs containing phosphorylated tau or aggregated α‐synuclein promote proteopathic spread, neuronal stress, and chronic neuroinflammation, recapitulating age‐related neuropathology [86, 87].

Although EVs are well‐established mediators of paracrine‐associated senescence, migrasomes represent a mechanistically distinct pathway by which MLCs propagate senescence. EVs are nanosized vesicles (30‐150 nm) generated through endosomal sorting or membrane budding and mainly deliver regulatory molecules such as miRNAs and cytokines that fine‐tune gene expression in recipient cells. In comparison, migrasomes are large vesicular organelles (0.5–3.0 μm) formed on retraction fibers during cell migration, capable of encapsulating and releasing bulk cytoplasmic material, including proteins, RNAs, and damaged organelles. Recent in vivo evidence demonstrates that migrasomes from senescent‐like BAMs induce paracrine senescence in neighboring microglia and accelerate cognitive decline, highlighting their functional relevance in brain aging [88].

Collectively, EVs and migrasomes establish a feed‐forward loop in which senescent MLCs convert surrounding cells into secondary senescent emitters, thereby amplifying chronic neuroinflammation and tissue dysfunction. Interfering with vesicle biogenesis, cargo loading, or migrasome formation attenuates paracrine senescence both in vitro and in vivo, underscoring their causal role and therapeutic potential in brain aging [89].

## 4 Heterogeneous Contribution of MLCs Subgroups to Brain Aging and Aging‐Associated Diseases

This section delineates the heterogeneous yet interrelated contributions of distinct MLC subsets to brain aging and age‐related diseases. We first examine how senescence reprograms microglia, BAMs, and infiltrating MDMs into maladaptive states and ultimately leads to a breakdown in neuroimmune equilibrium and communication across neural and vascular compartments. Finally, we highlight unresolved questions and emerging directions that may clarify how the coordinated dysfunction of MLC subsets shapes the trajectory of brain aging.

### 4.1 Contribution of Senescent MLCs to Brain Aging and Aging‐Associated Diseases

#### 4.1.1 Contribution of Senescent Microglia

Senescent microglia develop a characteristic functional imbalance, marked by heightened inflammatory output, loss of immunotrophic support and impaired maintenance of neuronal circuits. They overexpress inflammatory mediators, but show reduced expression of genes essential for debris clearance and neurotrophic support [90, 91]. This imbalance is further exemplified by a pathological shift in phagocytic priority. While their capacity for homeostatic surveillance and debris clearance declines, microglia exhibit an over‐pruning of functional synapses. This aberrant process is largely driven by the age‐related reactivation of the complement cascade, specifically the deposition of C1q and C3 on synapses, which tags them for excessive engulfment [18]. Such dysregulated synaptic refining, as a manifestation of senescent functional remodeling, contributes to the synaptic loss and cognitive decline characteristic of neurodegenerative diseases.

Increasing evidence links senescent microglia to the onset and progression of multiple age‐related neurodegenerative disorders. Microglial populations exhibiting features associated with senescence, including elevated p16^INK4a^ and p21 expression, a pro‐inflammatory cytokine profile resembling SASP, and transcriptional overlap with DAMs, are markedly enriched in aged brains and in regions most affected by neurodegeneration. Regardless of whether these cells are classified as truly senescent or chronically activated, their presence in aged brains and their correlation with neuropathology underscore their functional importance in brain aging [90, 92].

Microglial senescence is governed by intrinsic genetic factors. As mentioned earlier, *Trem2* and *Apoe4* are essential for microglial phagocytic and metabolic functions. Genetic variants in these genes further underscore their importance in maintaining microglial homeostasis. Mutations in *Trem*, such as the R47H variant, compromise microglial ability to clear Aβ and apoptotic cells, which leads to lipid metabolism dysfunction and exacerbates senescence [93, 94]. Clinical data show that *Trem2* R47H mutations significantly increase susceptibility to both dementia and PD. Similarly, *Apoe4*, the major genetic risk factor for late‐onset AD, impairs cholesterol metabolism in microglia. Compared to the *Apoe*3 allele, *Apoe*4 decreases cholesterol efflux, resulting in lipid droplet accumulation and increased oxidative stress, which promote microglial senescence [95, 96].

#### 4.1.2 Contribution of Senescent BAMs

Physiologically, BAMs maintain fluid homeostasis and clear metabolic waste via MMPs‐dependent ECM remodeling and phagocytosis. They also preserve NVU integrity through trophic factors that support vascular stability and neuronal survival. However, senescent BAMs compromise these functions, impairing glymphatic clearance and depriving the NVU of essential trophic support, thereby accelerating age‐related cognitive decline. With aging, BAMs exhibit reduced MMPs secretion, downregulated phagocytic genes, and decreased production of immunotrophic factors, impairing perivascular space integrity and toxic solute clearance [23, 97]. These deficits slow CSF‐ISF exchange, promote Aβ accumulation, disrupt metabolic homeostasis, and remove critical survival signals that deprive the NVU of essential trophic support, establishing a vicious cycle that exacerbates neuronal dysfunction and synaptotoxicity in AD and related dementias.

Senescent BAMs also erode CNS immune privilege. Under physiological conditions, BAMs enforce immune quiescence by constraining leukocyte trafficking at meningeal and perivascular interfaces, clearing antigens, and secreting anti‐inflammatory mediators to suppress local immune activation. As BAMs get senescent, these regulatory functions deteriorate, permitting infiltration of peripheral immune cells into the CNS. The consequent loss of immune containment amplifies neuroinflammation, disrupts neuronal integrity, and fosters the progression of neurodegenerative disorders [24].

#### 4.1.3 Contribution of Senescent CNS‐Infiltrating MDMs

Senescent MDMs propagate a self‐reinforcing cycle of inflammation and cellular senescence that progressively undermines CNS homeostasis during aging. The cascade originates in the periphery, where circulating monocytes, typically quiescent immune sentinels, undergo epigenetic reprogramming in response to chronically elevated inflammatory mediators. Following CNS infiltration, these epigenetically primed monocytes differentiate into senescent macrophages, acquiring a functionally deleterious phenotype characterized by sustained pro‐inflammatory signaling, markedly impaired phagocytic capacity, and compromised production of neuro‐supportive factors. Consequently, the failure to clear metabolic waste, together with the loss of trophic support and persistent neuroinflammatory signaling exacerbates neuronal injury [98]. Clinically, peripheral monocyte priming in PD patients correlates with disease severity, validating the pathogenic significance of this peripheral‐central inflammatory axis [99].

### 4.2 Unsolved Questions and Future Research Directions

#### 4.2.1 Unsolved Questions Associated With Senescent Microglia

##### 4.2.1.1 Downregulation of Neuroprotective Functions

Marco Prinz and colleagues have shown that microglia contribute to neuronal glycolipid metabolism by delivering β‐hexosidase B (HEXB), a process vital for neuronal health [100]. Despite these findings, the precise mechanisms through which aging modulates this function remain unclear. Specifically, do senescence‐related signaling pathways like p53 influence the synthesis and secretion of HEXB and other protective factors? Furthermore, how does aging impact the transport efficiency of these critical substances? Answering these questions may open up new avenues for therapeutic strategies aimed at restoring microglial neuroprotection in age‐related diseases.

##### 4.2.1.2 Propagation of Senescent Organelles

Michael T. and colleagues have shown that healthy microglia can deliver mitochondria to neurons through tunneling nanotube (TNT)‐mediated transfer, aiding mitochondrial repair in dementia models [101]. However, whether senescent microglia can similarly transfer organelles to other cells remains to be determined. What are the effects of organelles from senescent cells on their recipient counterparts, and could this process itself propagate senescence? Clarifying the role of organelle transfer in senescence spread could offer new avenues for therapeutic intervention to block its transmission in aging and neurodegenerative disorders.

##### 4.2.1.3 Region‐Specific Aging Characteristics

Sean C. Bendall and colleagues have revealed that microglia show significant heterogeneity across different brain regions, including the hippocampus, cortex, and cerebellum [102]. However, it remains to be clarified how these regional differences in microglial aging contribute to specific aging phenotypes. For example, is microglial aging in the hippocampus particularly associated with impairments in learning and memory? Understanding the regional specificity of microglial aging could form the foundation for more targeted interventions aimed at alleviating age‐related cognitive decline.

#### 4.2.2 Unsolved Questions Associated With Senescent BAMs

A pivotal question concerns how aging reprograms the functional plasticity of BAMs. Whereas BAMs adopt a reparative phenotype that facilitates tissue recovery after acute CNS injury, evidence suggests that this capacity is compromised with age [25, 97]. We hypothesize that aging induces a maladaptive phenotypic shift, transforming BAMs from regenerative actors into drivers of chronic inflammation. This model predicts that aged BAMs overexpress chemokines, promoting dysregulated monocyte infiltration, and that molecular drivers, including epigenetic reprogramming or a sustained SASP, lock them into this deleterious state. Elucidating the mechanisms underlying this functional transition is essential for developing targeted therapies to reinstate BAMs‐mediated repair in the aging CNS.

A second unresolved question is whether senescent BAMs function as bidirectional relays for senescence signals across the CNS‐periphery interface. We hypothesize that BAMs‐derived migrasomes or soluble SASP transit meningeal lymphatic vessels to disseminate proaging signals systemically, potentially influencing peripheral organs such as the bone marrow and spleen. Conversely, an equally critical hypothesis posits that BAMs detect peripheral inflammatory or senescent cues and relay them into the brain parenchyma, which might involve specialized meningeal transport mechanisms. Establishing the existence and regulation of this bidirectional circuit will clarify the role of BAMs in system‐wide aging and reveal novel targets for blocking pathological signal propagation.

A third critical uncertainty lies in whether the loss of BAM‐mediated immunotrophic support acts as a primary driver or a secondary casualty of NVU collapse. While it is believed that BAMs produce essential factors to maintain vascular patency [103], it remains unclear whether aging induces a trophic‐silencing state. In this state, BAMs may persist numerically but become molecularly incompetent, failing to sustain protein synthesis due to metabolic exhaustion. Furthermore, the bioenergetic trade‐off within senescent BAMs remains largely unexplored: does the high metabolic demand of maintaining a chronic SASP‐secreting state shunt ATP away from the biosynthesis of neuroprotective trophic factors? Deciphering whether this trophic dereliction is reversible through metabolic reprogramming, such as by targeting mitochondrial efficiency or lipid droplet accumulation, could offer a transformative strategy for restoring the structural‐to‐functional stability of the aging brain.

#### 4.2.3 Unsolved Questions Associated With Senescent MDMs

A key unresolved question is whether perivascular monocyte‐derived cells act as messengers that transmit brain aging signals to the periphery, thereby perpetuating a self‐reinforcing cycle of systemic and brain aging. This concept is supported by evidence that meningeal lymphatic vessels drain CNS contents to cervical lymph nodes, where they shape peripheral immunity [104, 105]. Whether such peripherally‐exposed monocytes acquire a pro‐inflammatory phenotype and subsequently impact the CNS remains an open question. Building on our previous finding that MDMs produce vasculopathic migrasomes [106], we hypothesize that senescence converts these cells from local protectors into systemic broadcasters of senescence signals that fuel this vicious cycle. Several critical gaps in our understanding remain: What signals initiate this cellular reprogramming? What is the molecular nature of the propagated senescence cargoes? How do these systemic signals, in turn, feed back to the brain? Addressing these questions is essential for identifying novel therapeutic targets capable of disrupting this detrimental feedback loop in age‐related neurodegeneration.

## 5 Therapeutic Strategies Targeting MLCs Aging

Given the critical roles of MLCs in brain aging, we aim to summarize therapeutic strategies targeting these cells. This section discusses current and emerging interventions designed to restore MLCs' function and resilience, providing a framework for preserving neuroimmune integrity in the aging brain.

### 5.1 Current Therapeutic Strategies

#### 5.1.1 Senolytics: Direct Removal of Senescent MLCs

Senolytics selectively eliminate senescent cells through inhibition of their characteristic antiapoptotic pathways [107]. The elimination of senescent cells is therapeutically pivotal because they chronically destabilize tissue homeostasis. By concurrently evading apoptosis, suppressing regeneration, and sustaining inflammation, they drive a self‐reinforcing cycle of decline. Senolytics intervene by resetting these pathogenic circuits.

The most compelling evidence to date involves the dasatinib and quercetin (D + Q) combination, which acts through a multitarget mechanism: dasatinib inhibits prosurvival kinases such as proto‐oncogene tyrosine‐protein kinase Src (SRC) and proto‐oncogene tyrosine‐protein kinase (ABL), while quercetin antagonizes the antiapoptotic proteins B‐cell lymphoma 2 (BCL‐2) and B‐cell lymphoma‐extra large (BCL‐XL), collectively triggering apoptosis [108]. In models of AD and in clinical cohorts, D + Q treatment clears senescent cells including MLCs, enhances Aβ clearance, and improves cognitive performance [109, 110].

Other senolytic agents further illustrate the ongoing pursuit of target specificity. For instance, the BCL‐2 pathway inhibitor ABT‐263 effectively depletes senescent cells but is limited by adverse effects such as thrombocytopenia [111, 112]. In contrast, FOXO4‐DRI operates through a distinct mechanism by disrupting the FOXO4‐p53 interaction, thereby promoting p53‐mediated apoptosis in senescent cells while demonstrating a favorable safety profile with no significant peripheral toxicity [113].

Despite these promising results, the clinical translation of senolytics faces challenges. First, most compounds exhibit poor BBB penetration, restricting their delivery to the CNS and necessitating advanced strategies such as nanocarriers or localized administration. Second, the current inability to accurately distinguish senescent from normal MLCs subpopulations raises the risk of off‐target cell loss and potential disruption of CNS immune homeostasis. Addressing these limitations may require novel approaches. One promising direction might involve integrating single‐cell sequencing and structural biology to inform the design of next‐generation senolytics. This strategy might offer a path toward improved brain accessibility and cellular specificity, which will be essential for achieving an optimal balance between treatment efficacy and safety in aging and neurodegenerative contexts.

#### 5.1.2 Senomorphics: Inhibiting Senescence Propagation Rather Than Clearing Cells

Unlike senolytics, senomorphic agents modulate, rather than eliminate, senescent cells. This approach may offer a superior therapeutic window by preserving post‐mitotic cells like neurons and maintaining tissue homeostasis, thereby avoiding the risks of atrophy associated with cell clearance [114, 115].

Senomorphic strategies primarily operate through two complementary mechanisms: (1) Suppression of prosenescence mediator production through targeting overactivated innate immune signaling pathways. Representative strategies include cGAS‐STING inhibitors (e.g., PF‐06928215 and H‐151), NLRP3 inflammasome inhibitors (e.g., MCC950), and NF‐κB pathway blockers (e.g., curcumin), which collectively attenuate key pro‐inflammatory cytokines such as IL‐1β, TNF‐α, and IL‐6. These interventions have been shown to ameliorate neuroinflammation and restore cognitive function across multiple aging models [116, 117, 118]. (2) Interception of prosenescence signal dissemination by neutralizing extracellular mediators of senescence. This includes the use of cytokine‐specific neutralizing antibodies, such as siltuximab targeting IL‐6 and adalimumab against TNF‐α, which effectively alleviate systemic inflammation [119, 120]. More recently, targeting migrasomes, specialized vesicles that transport senescence‐promoting cargos like AIM, has emerged as a promising strategy to disrupt the intercellular propagation of senescent phenotypes [88].

Despite these advances, several challenges impede the clinical translation of senomorphic therapies. First, the spectrum of senescence‐promoting mediators is highly diverse and extends well beyond the classical SASP, complicating the identification of comprehensive therapeutic targets. Second, there is considerable interindividual heterogeneity in the core signaling networks that drive the senescence program; while some individuals exhibit a reliance on NF‐κB, others may depend more on STAT or CD5L‐related pathways, underscoring the need for personalized therapeutic approaches. Third, significant practical hurdles remain, including uncertainties regarding long‐term safety, suboptimal specificity in drug delivery to senescent cell populations, and a limited understanding of the distinct functional roles of various extracellular vesicles, such as migrasomes and exosomes. One promising strategy to address these challenges involves leveraging multiomics to define individual senescence signatures, advancing targeted delivery systems, and assessing the long‐term benefits of personalized senomorphic regimens.

#### 5.1.3 Metabolic Intervention: Repair MLCs Metabolic Imbalance

Metabolic interventions aim to counteract aging by restoring bioenergetic and redox homeostasis. In the aging brain, glucose hypometabolism triggers a vicious cycle of energetic deficit that amplifies cumulative damage via synergistic toxicity from lipid peroxidation, protein aggregation, and iron‐mediated oxidative stress [73, 121]. Therapeutic strategies accordingly seek to rectify dysregulation across these core pathways to reestablish metabolic balance.

Evidence comes from CNS‐focused studies: intranasal insulin and GLP‐1 agonists such as liraglutide attenuate tau hyperphosphorylation and microglial activation, thereby countering neurodegeneration [122]; peroxisome proliferator‐activated receptor γ (PPARγ) activation by pioglitazone attenuates neuroinflammation in experimental models [123]; heat shock protein 90 (HSP90) inhibitors and mechanistic target of rapamycin (mTOR) modulators reduce protein aggregation and restore proteostasis [124, 125] and iron chelators such as deferoxamine alleviate iron‐induced oxidative stress and promote repair [126]. Together, these strategies illustrate the potential of multitargeted metabolic interventions to disrupt the self‐reinforcing cycle of brain aging. However, it is crucial to distinguish direct CNS effects from systemic benefits. Although some of these approaches do not selectively target MLCs, they are likely to combine direct signaling with systemic metabolic improvements to alleviate the metabolic and oxidative burden that drives MLCs dysfunction, thereby indirectly supporting their homeostatic and neuroprotective functions. Although many metabolic interventions exert systemic effects that secondarily benefit the brain, direct CNS‐specific bioenergetic rescue is supported by FDG‐PET imaging, which reveals a persistent glucose deficit in the aging brain that can be mitigated by alternative fuels such as ketones [73, 127, 128]. While systemic metabolic improvements, such as enhanced insulin sensitivity, may support brain health, evidence for their direct CNS effects remains largely indirect, and conclusions should be drawn accordingly.

Clinical translation, however, is hampered by limited BBB penetration, restricting intracranial bioavailability and efficacy. Future work could explore brain‐penetrant metabolic modulators delivered via nanocarriers, BBB‐shuttling peptides, or by leveraging the innate phagocytic capacity of MLCs to enhance targeted delivery. These interventions could be combined with senolytics to eliminate resistant senescent MLCs or with organelle‐targeted therapies to restore mitochondrial and lysosomal function. Such integrated strategies may support MLCs' homeostasis and regenerative capacity while providing coordinated, multilevel antiaging benefits.

#### 5.1.4 Lifestyle Adjustment: The Multifaceted Modulator of Delaying Aging

Lifestyle interventions modulate brain aging by systemically regulating metabolism, inflammation, and neuroendocrine networks. As safe and economical alternatives to pharmacological approaches, they act through energy metabolism reprogramming and oxidative stress control to prevent age‐related cognitive decline.

The most direct CNS evidence comes from nutritional ketosis. Clinical studies have shown that daily intake of medium‐chain triglycerides (MCTs) doubles brain ketone uptake and significantly improves executive function and memory in patients with mild cognitive impairment (MCI) [73], indicating that ketones can directly fuel cerebral metabolism. For other interventions, such as caloric restriction, exercise, intermittent fasting, adequate sleep, and balanced nutrition, evidence largely derives from systemic metabolic improvements, including enhanced insulin sensitivity, reduced peripheral inflammation, and lower inflammatory signals such as SASP [129, 130, 131]. The extent to which these systemic benefits translate to CNS effects, and specifically to MLC function, is still under investigation. These integrated lifestyle strategies, potentially alleviating the metabolic and inflammatory burden on MLCs, offer a powerful approach to protecting the brain during aging.

However, several challenges remain in optimizing these interventions. Future studies are needed to establish precise dose–response relationships and intervention durations through large‐scale clinical trials, elucidate underlying mechanisms using multiomics approaches, and explore synergistic applications with pharmacological and cellular therapies, with the goal of developing comprehensive multidimensional intervention systems for MLCs senescence and brain aging.

### 5.2 Emerging Therapeutic Strategies

#### 5.2.1 Cell Replacement and Transplantation Therapy

Besides senolytics, a compelling strategy involves the wholesale replacement of the senescent microglial population with rejuvenated cells. This approach seeks to directly address age‐related microglial dysfunction, which is characterized by deficient waste clearance, chronic pro‐inflammatory activation, and aberrant synaptic connectivity. By substituting this deteriorated innate immune population, the therapy aims to rectify the primary cellular dysfunctions that perpetuate the cycle of neuroinflammation and neurodegeneration.

Pioneered by the microglia engineering toolkit for serially transplanting engineered repopulating microglia (MISTER) system [132], this approach enabled donor microglia to repopulate over 90% of the aged host niche in a colony stimulating factor 1 receptor (CSF1R) mutant model, restoring phagocytosis, synaptic pruning, and inducing a transcriptomic reversal of aging. Preliminary clinical data in an eight‐patient cohort further reported stabilized brain structure and improved neurological function over 2 years [133, 134]. The therapeutic potential of microglial replacement extends beyond a single disease entity, from AD [135] to lysosomal storage disorders [136], and highlights a universal mechanism whereby replenishment of the microglial compartment resets a dysregulated cerebral immune milieu and mitigates aging‐associated pathology [137, 138]. Although not primarily an antisenescence therapy, microglia replacement fundamentally addresses the aged, senescent cell‐laden tissue environment that drives pathology. It thus represents a transformative strategy to potentially delay or prevent a spectrum of age‐related neurological disorders.

Several questions remain to be addressed for the translational progression of this approach. Key steps would include expanding clinical evaluation to a broader range of age‐related neurological conditions, which would help evaluate its potential applicability even in the context of nonpathological aging. Meanwhile, the refinement of cell sources, such as induced pluripotent stem cell (iPSC)‐derived or engineered microglia, could enable more tailored regimens in the future. Furthermore, long‐term studies are warranted to determine the durability of the microglial rejuvenation and its sustained effects on brain immune homeostasis. Addressing these aspects would be valuable for further assessing the translational potential of microglia replacement.

#### 5.2.2 Organelle Renewal Therapy: Targeting the Initiation of MLCs Aging

Organelle renewal therapy aims to restore functional organelle states in aging MLCs, targeting the source of MLCs senescence. Manifestations of senescence (e.g., reduced phagocytosis, SASP) often originate from the failure of core organelles, making their functional restoration a direct path to reversing cellular decline. The lysosome‐mitochondria axis represents a key target, where lysosomal decline triggers mitochondrial dysfunction via ROS and impaired mitophagy, establishing a self‐reinforcing vicious cycle. This core dysfunction is amplified by the collapse of the broader proteostatic network, where failures in the ubiquitin‐proteasome system and LC3‐associated phagocytosis (LAP) exacerbate the burden on lysosomes and intensify neuroinflammation.

Interventions targeting this dysfunctional network have shown promise. A key strategy for lysosomal renewal is transcription factor EB (TFEB) activation. The natural disaccharide trehalose, for instance, enhances lysosomal function and promotes Aβ clearance in aged mice [139, 140]. Concurrently, mitochondrial renewal via enhanced mitophagy is achieved with compounds like Urolithin A that improve function and reduce neuroinflammation in aged microglia [141]. Together, these findings underscore the therapeutic potential of targeting the lysosome–mitochondria–proteostasis network.

While the mechanistic rationale is compelling, key translational challenges remain. The current therapeutic pipeline consists predominantly of preclinical candidates, requiring further elucidation of their long‐term safety and pharmacokinetic profiles, particularly regarding BBB penetration and cell‐type specificity. Future efforts entail developing optimized CNS‐penetrant modulators and exploring synergistic combinations between lysosomal and mitochondrial renewal pathways. Exemplified by the novel migrasome‐dependent mitochondrial quality control pathway revealed by Li Yu [142], augmenting intracellular repair by stimulating endogenous clearance mechanisms constitutes a promising complementary frontier. A longitudinal evaluation of intervention durability and homeostatic impact in physiologically aged contexts will be critical.

#### 5.2.3 Phagostasis Therapy: Balance Phagocytosis Function and Resistance to Aging

Phagostasis therapy aims to enhance endogenous protective mechanisms in MLCs. This approach addresses a fundamental biological dilemma: although MLCs are essential for clearing neurotoxic waste such as Aβ, this very function inflicts damage upon the cells themselves. In youth, MLCs maintain a delicate equilibrium through efficient self‐repair mechanisms; however, aging disrupts this balance, resulting in cumulative cellular damage and the onset of senescence [57]. The goal of phagostasis therapy is to restore this compromised homeostasis in MLCs by reinforcing critical cytoprotective pathways.

A key strategy involves activating the nuclear factor erythroid 2‐related factor 2 (NRF2) antioxidant pathway to counter oxidative stress from phagocytosis. For example, sulforaphane, which inhibits Kelch‐like ECH‐associated protein 1 (KEAP1), has been shown to reduce oxidative damage in aged mice [143, 144]. Similarly, supporting the heat shock response can help manage protein‐folding stress under phagocytic load. Drugs like 17‐AAG, which inhibit HSP90, work by activating the transcription factor heat shock factor 1 (HSF1). This enhances the function of protein‐folding assistants and reduces proteostatic stress in age‐related disease [145].

While these initial findings are encouraging, they also highlight several key challenges. The translation of this strategy will require the development of CNS‐optimized activators that exhibit improved cell‐type specificity, coupled with rigorous long‐term safety evaluations. Furthermore, exploring potential synergies with complementary approaches, such as senolytic therapies, represents a logical next step for enhancing efficacy against age‐related neurodegenerative pathologies.

**FIGURE 4:**
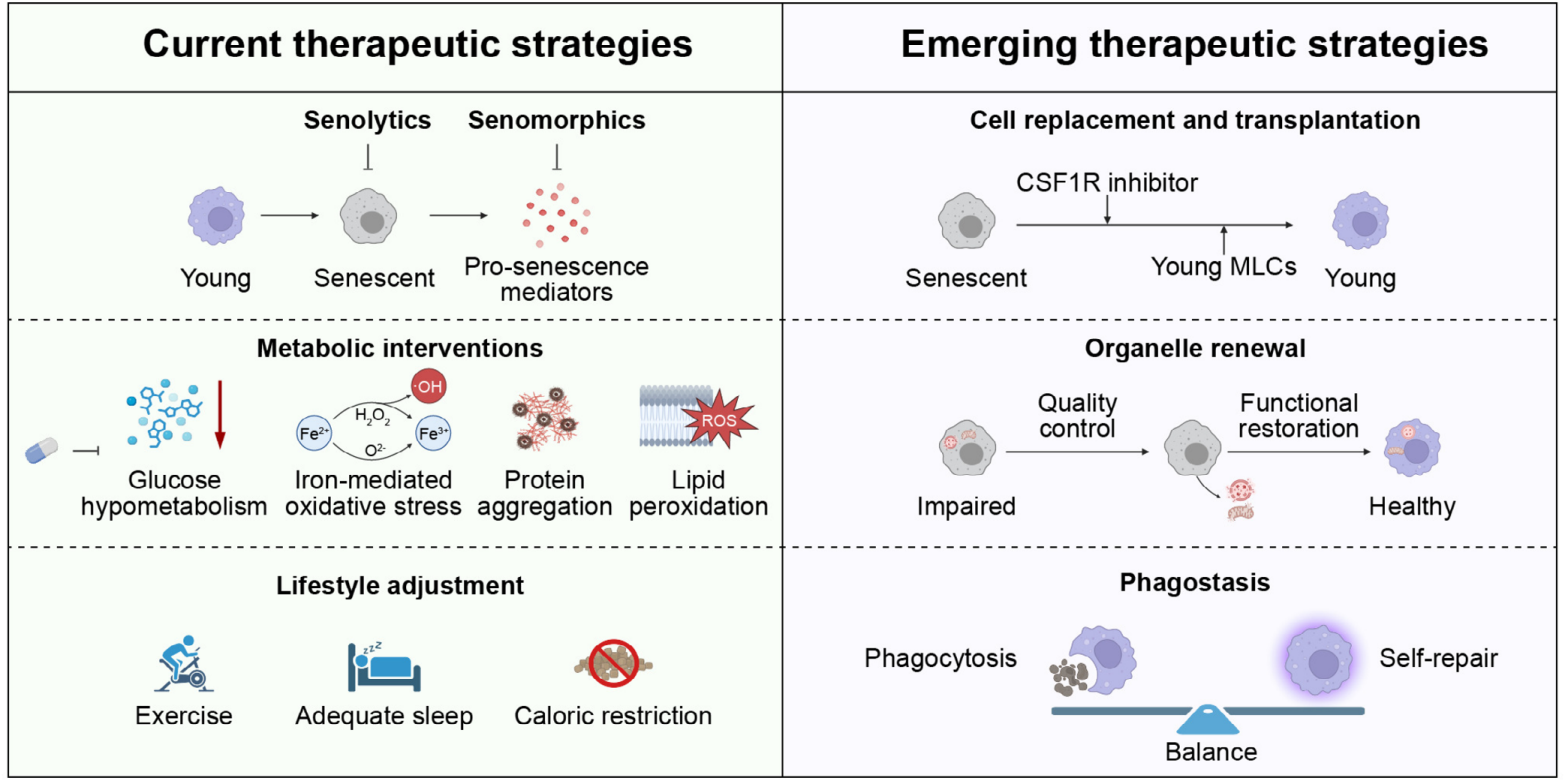
Current and emerging therapeutic strategies targeting MLCs senescence. Current therapeutic strategies to counteract MLCs senescence encompass senolytics for cell clearance, senomorphics to suppress the production and dissemination of pro‐senescence mediators, metabolic interventions, and lifestyle adjustment. Emerging approaches include cell replacement and transplantation, organelle renewal, and phagostasis therapy, all aimed at restoring MLCs homeostasis and function.

Together, these strategies reflect a shift from merely eliminating senescent MLCs to restoring their regenerative competence, underscoring that rejuvenating immune resilience rather than depletion alone may hold the key to preserving neuroimmune integrity with age (Figure 4, Table 2).

**TABLE 2:** Therapeutic strategies targeting MLCs senescence.

Strategy	Subtype	Mechanism of action	Evidence stage	BBB penetration	Major risks/adverse effects
Senolytics	Dasatinib + Quercetin (D + Q)	Dasatinib inhibits SRC/ABL kinases; Quercetin inhibits multiple pro‐survival pathways; together eliminating senescent cells	Clinical	Good (CNS penetration)	Cough, nausea, and headaches; mild gastrointestinal (GI) symptoms; QT prolongation not reported at senolytic doses (risk at oncologic doses)
	ABT‐263 (Navitoclax)	Inhibits BCL‐2/BCL‐xL to induce apoptosis in senescent cells	Animal for aging/fibrosis	Poor (Active efflux by P‐glycoprotein)	Dose‐limiting thrombocytopenia
	FOXO4‐DRI	Disrupts FOXO4‐p53 interaction to promote p53‐mediated apoptosis in senescent cells	Animal	Unknown	Protective in most aging models; detrimental in pulmonary hypertension; long‐term safety in humans unknown.
Senomorphics	cGAS‐STING inhibitors (H‐151)	H‐151 covalently binds STING (Cys91) to block its activation and TBK1‐IRF3 signaling	Animal	Good (CNS activity in animals)	Long‐term safety unknown; potential immunosuppression
	NLRP3 inhibitors (MCC950)	Blocks inflammasome activation, reducing IL‐1β/IL‐18 production	Animal	Good (CNS activity in animals)	Chronic immunosuppression risk; hepatotoxicity at high doses
	NF‐κB inhibitors (curcumin)	Inhibits pro‐inflammatory cytokine transcription and activates NRF2 antioxidant pathway	Animal	Poor (High metabolic turnover; P‐glycoprotein substrate; nano‐formulations under study)	Mild GI symptoms; occasional headaches
	Cytokine‐neutralizing antibodies (siltuximab, adalimumab)	Neutralizes extracellular IL‐6 (siltuximab) or TNF‐α (adalimumab)	Clinical (not tested for CNS senescence)	Poor (no meaningful BBB penetration)	Systemic immunosuppression; increased infection risk; paradoxical inflammatory syndrome
	Migrasome‐targeting	Blocks intercellular propagation of senescent signals via migrasomes	Animal	Not established	Requires further mechanistic validation
Metabolic interventions	Intranasal insulin	Enhances cerebral glucose uptake and improves neuronal energy metabolism	Clinical	Excellent (bypasses BBB via intranasal route)	Nasal irritation; hypoglycemia risk minimal at 40 IU dose
	GLP‐1 agonists (liraglutide)	Improves cerebral glucose metabolism, reduces neuroinflammation, and affects MLCs indirectly	Clinical	Limited	Gastrointestinal (nausea, vomiting); long‐term CNS effects unclear
	PPARγ agonists (pioglitazone)	Attenuates lipid peroxidation, reduces neuroinflammation, and improves insulin sensitivity	Clinical	Good (CNS penetrant)	Edema; weight gain; fracture risk; fluid retention; heart failure risk
	mTOR modulators (rapamycin)	Restores proteostasis, modulates MLC metabolism, and enhances autophagy	Clinical	Moderate to Good (Dose‐dependent CNS penetration)	Immunosuppression; metabolic disturbances
	Iron chelators (deferiprone)	Reduces iron‐induced oxidative stress and ferroptosis	Clinical approved for thalassemia; animal for neurodegeneration	Limited	Agranulocytosis; GI symptoms
Lifestyle interventions	Nutritional ketosis (MCTs)	Provides ketones as alternative brain fuel	Clinical	Good (ketones cross BBB)	GI discomfort (diarrhea, bloating); long‐term adherence challenging
	Caloric restriction/Intermittent fasting	Improves systemic metabolism and reduces inflammation	Clinical	Indirect (systemic only)	Malnutrition risk; not suitable for frail elderly; hypoglycemia risk in diabetics
	Exercise	Enhances BDNF, vascular function, and systemic anti‐inflammatory effects	Clinical	Indirect (peripheral‐mediated)	Injury risk; requires sustained adherence; benefits vary by intensity/duration
Cell replacement	Microglia replacement	Transplants donor microglia to repopulate aged CNS niche	Animal; clinical (pilot human cohort, *n* = 8)	N/A (cells delivered directly via transplantation)	Pre‐conditioning toxicity (CSF1R inhibitor‐mediated niche clearance); genomic stability of donor cells; tumorigenic potential; immune rejection; ethical considerations; long‐term safety unknown
Organelle renewal	TFEB activators (trehalose)	Enhances lysosomal function and autophagy	Animal	Limited	GI intolerance at high doses
	Mitophagy enhancers (Urolithin A)	Promotes mitochondrial renewal and reduces microglial neuroinflammation	Clinical	Good (BBB penetrate)	Generally well‐tolerated in peripheral human studies
Phagostasis therapy	Sulforaphane	Activates antioxidant pathways to counter oxidative stress	Animal	Good (crosses BBB)	Generally safe (dietary compound from broccoli); limited human CNS data
	17‐AAG	Induces heat shock response via HSP90 inhibition	Animal	Poor	Hepatotoxicity; myelosuppression at high doses; limited therapeutic window; poor BBB penetration limits first‐generation variants; second‐generation analogs (e.g., Ganetespib) under CNS investigation

## 6 Perspectives and Conclusions

### 6.1 Basic Research: Deciphering the Specificity, Heterogeneity and Interaction Mechanisms of MLCs Senescence

The path toward precise interventions for senescent MLCs presents challenges, many of which are rooted in a few fundamental areas: the current lack of specific biomarkers, still unclear spatial and functional links, and as yet undefined intercellular communication mechanisms. Addressing these questions will require a concerted research effort.

Current definitions of MLCs senescence heavily rely on universal markers like SASP, which lack specificity for distinct subpopulations or senescence stages. The application of multiomics technologies is therefore of considerable value for identifying unique molecular signatures, such as those marking early‐stage microglia or functionally impaired BAMs, to enable precise detection and targeting. Beyond identification, the field would benefit from establishing causal links between region‐specific MLCs senescence and local functional decline. For instance, determining how senescent hippocampal microglia contribute to circuit disruption and memory loss would provide a rationale for developing spatially precise therapies that minimize off‐target effects. Furthermore, understanding how senescence propagates requires elucidating key intercellular communication pathways. Deciphering mechanisms like migrasome‐mediated signaling from BAMs to microglia, alongside exploring dynamics such as functional compensation between central and peripheral MLCs pools, will reveal strategic nodes for blocking the spread of senescence signals.

Collectively, research focused on specific biomarkers, spatial‐functional mapping, and intercellular communication will be helpful to establish the foundational framework for precise intervention in MLCs‐driven brain aging.

### 6.2 Technology‐Driven Transformation: Overcoming Specificity and Safety Hurdles in MLCs‐Targeted Therapy

While current intervention strategies hold therapeutic promise, their translation into precise and safe clinical applications for MLCs faces considerable challenges. Advancing the field may require an integrated technological framework that addresses delivery efficiency, cellular specificity, and long‐term safety.

Enhancing delivery efficiency to brain MLCs is critical, requiring carriers that target MLCs‐specific surface receptors, thereby improving BBB penetration and cellular uptake to achieve therapeutic intracerebral concentrations of senolytics and organelle modulators. Beyond delivery, therapeutic strategies should account for the functional heterogeneity of MLCs. Future approaches could move beyond uniform intervention by employing cell‐type‐specific actions. For instance, a therapeutic approach could involve applying senolytics to detrimental populations while using functional modulators on other subsets, thereby preserving overall CNS immune competence. Concurrently, comprehensive safety assessment is imperative. In addition to evaluating short‐term efficacy, future studies should rigorously monitor the long‐term impacts of these interventions on brain immune surveillance and neural repair functions. Critical evaluations include assessing the genomic stability of engineered donor cells and the potential risks associated with prolonged senolytic administration.

Collectively, parallel progress in targeting, specificity, and safety profiling will pave the way for a new generation of precise and viable MLCs‐directed therapies for age‐related brain disorders.

### 6.3 Clinical Application: Construct a Multidimensional Brain Aging Management System With MLCs as the Core

Future brain aging management necessitates a shift from singular interventions toward integrated, multidimensional systems. Within such a framework, MLCs emerge as a pivotal biological nexus, orchestrating multiple age‐related processes. The clinical translation of this concept unfolds along two complementary avenues: the incorporation of MLCs‐derived biomarkers into diagnostic algorithms and the rational design of MLCs‐informed combination therapies.

In the diagnostic realm, senescence‐associated signatures of MLCs hold potential as tools for early detection and dynamic monitoring. CSF analysis, for instance, quantifying specific SASP factors or migrasomes shed by senescent BAMs, could provide a window into glymphatic function and the initial stages of cellular senescence. Concurrently, advanced imaging modalities, such as MLCs‐specific positron‐emission tomography (PET) tracers, are being developed to visualize the spatiotemporal progression of these aging processes in vivo. Together, these approaches could refine disease staging and improve prognostic accuracy. Therapeutically, a stratified approach based on disease progression appears promising. In asymptomatic at‐risk populations, primary interventions might combine lifestyle modifications with metabolic modulators to delay senescence onset. In cases of mild cognitive impairment, a dual‐action approach combining PPARγ agonists with NLRP3 inhibitors may offer a promising means to simultaneously address metabolic decline and neuroinflammation. In advanced AD, strategic cell replacement therapies, potentially augmented with TFEB agonists to bolster phagocytic clearance, might offer a path to restore CNS homeostasis.

Collectively, the full realization of this MLCs‐centric strategy will be propelled by continued elucidation of underlying mechanisms, refinement of targeted delivery technologies, and rigorous clinical validation, ultimately contributing to novel, holistic strategies for preserving brain health with advancing age.

In summary, this Review underscored the central roles of MLC subsets in brain aging. Their senescence, driven by phagocytic overload and metabolic stress, disrupts homeostatic functions and promotes neuroinflammation, thereby accelerating the pathogenesis of age‐related neurodegenerative diseases such as AD and PD. While these subsets form the core of the CNS MLCs network, it is crucial to recognize their interplay with other immune and glial cells. Astrocytes and lymphocytes, which also undergo age‐related functional decline, engage in complex bidirectional communication with MLCs, collectively shaping the brain aging process. Furthermore, the impact of senescent MLCs is likely not confined to cognitive decline but may extend to other domains of brain health, including emotional regulation, sleep, and motor control, which warrants further investigation. Finally, systemic factors such as peripheral inflammation emerge as potent modulators of MLCs' senescence, particularly in frailty, underscoring the need to elucidate how the systemic environment influences brain aging.

## Author Contributions

Wei Cai, Zhengqi Lu, and Wei Qiu designed the concept and the idea of this article. Qin Qin, Liubin Zhang, and Manning Guo designed and wrote this manuscript and designed the picture and table. Danli Lu, Yuxin Liu, Zihong Wang, and Mengyan Hu contributed to the revision of the manuscript. Shisi Wang, Xinmei Kang, and Haotong Yi provided constructive advice and participated in proofreading. All authors contributed to the article and approved the submitted version. All authors have read and approved the manuscript.

## Funding

This work was supported by grants from the Guangdong Basic and Applied Basic Research Foundation (2023A1515012530 to Z.L.), the National Natural Science Foundation of China (82271348 to W.C.), the Guangdong Basic and Applied Basic Research Foundation (2024B1515020021 to W.C.).

## Ethics Statement

The authors have nothing to report.

## Consent

The authors have nothing to report.

## Conflicts of Interest

The authors declare no conflicts of interest.

## Data Availability

Data sharing is not applicable to this article as no new data were created or analyzed in this study.
